# Correction to “Overexpression of FBR41 Enhances Resistance to Sphinganine Analog Mycotoxin‐Induced Cell Death and Alternaria Stem Canker in Tomato”

**DOI:** 10.1111/pbi.70721

**Published:** 2026-07-17

Shao, Z., Y. Zhao, L. Liu, et al. 2020. “Overexpression of FBR41 Enhances Resistance to Sphinganine Analog Mycotoxin‐Induced Cell Death and Alternaria Stem Canker in Tomato.” *Journal of Plant Biotechnology* 18: 141–154. https://doi.org/10.1111/pbi.13182.

In the above article, the authors would like to correct the Acknowledgements from ‘This work was supported by the Ministry of Agriculture of China (2016ZX08009003‐001), National Natural Science Foundation of China (31200230, 31601746) and Zhejiang Provincial Natural Science Foundation of China (LZ15C150001).’ to ‘This work was supported by the Ministry of Agriculture of China (2016ZX08009003‐001), National Natural Science Foundation of China (31830078, 31200230, 31601746) and Zhejiang Provincial Natural Science Foundation of China (LZ15C150001).’

We apologise for this error.

